# Insights into the Origin of High Activity of Ni_5_P_4_(0001) for Hydrogen Evolution Reaction

**DOI:** 10.1021/acs.jpcc.3c00238

**Published:** 2023-03-09

**Authors:** Yang Yang, Xiao Lin, Yang Li, Tian Sheng, Shaoan Cheng, Xiaoming Sun, Wen-Feng Lin

**Affiliations:** † Department of Chemical Engineering, Loughborough University, Loughborough, Leicestershire LE11 3TU, United Kingdom; ‡ Department of Chemical Engineering and Biotechnology, University of Cambridge, Cambridge, CB3 0AS, United Kingdom; § College of Chemistry and Materials Science, Anhui Normal University, Wuhu, 241000, China; ∥ State Key Laboratory of Clean Energy, School of Energy Engineering, Zhejiang University, Hangzhou 310027, China; ⊥ State Key Laboratory of Chemical Resource Engineering, Beijing Advanced Innovation Center for Soft Matter Science and Engineering, College of Chemistry, Beijing University of Chemical Technology, Beijing, 100029, China

## Abstract

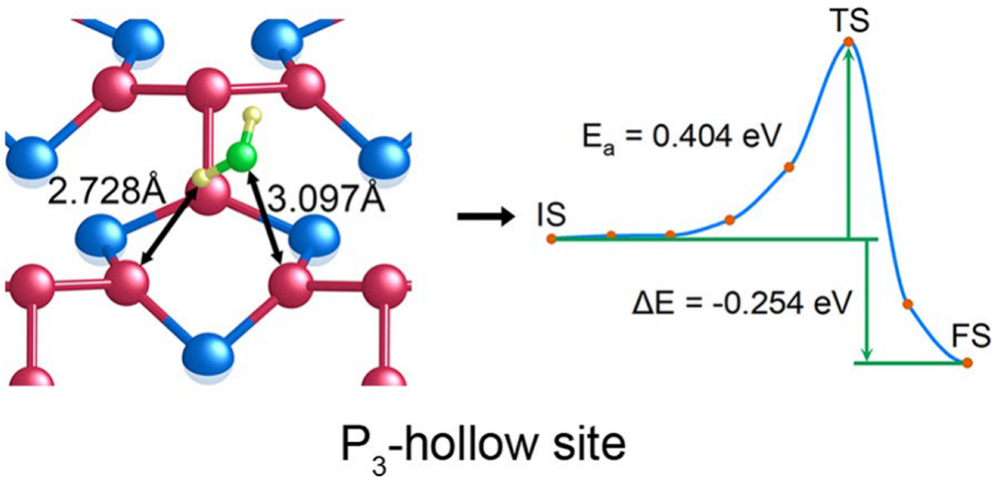

Hydrogen evolution reaction (HER) is directly relevant to green hydrogen production from water splitting. Recently, a low-cost Ni_5_P_4_ material has been demonstrated experimentally and theoretically to exhibit excellent electrocatalytic activity toward HER. However, a fundamental understanding of the origin of Ni_5_P_4_(0001) activity is still lacking. In this work, density functional theory (DFT) calculations were employed for a comprehensive investigation. The calculation results indicate that the Ni_5_P_4_(0001) surface exposing Ni_3_P_4_ termination gains the highest stability, on which a nearly thermoneutral hydrogen adsorption was found at the P_3_-hollow sites, providing a high activity for HER. The activity was also observed to be maintained over a wide H-coverage. HER occurs via the Volmer–Heyrovsky mechanism as evidenced from the optimal hydrogen adsorption free energy, but unlikely through the Tafel reaction due to its large energy barrier. Furthermore, the P_3_-hollow sites also exhibit a low kinetic barrier for water dissociation, promoting HER in alkaline media. A series of electronic structure analyses were performed in gaining insights into the origin of the HER activity. First, the density of states (DOS) and crystal orbital Hamilton population (COHP) analyses revealed a favorable interaction of electronic states between P and H atoms, leading to stable H adsorption at P_3_-hollow sites. In addition, the Bader charge analysis demonstrates that the strength of H adsorption at P_3_-hollow sites linearly increases with the electrons carried by the latter. The optimal net charge on the P_3_-hollow sites leads to a desired Δ*G*_H_ that is close-to-zero. Finally, a highly efficient electron transfer was observed between the P_3_-hollow sites and their neighboring atoms, facilitating the HER.

## 1 Introduction

Hydrogen can serve as the primary energy carrier of future energy systems due to its several distinct advantages such as high energy density and zero-carbon emission.^1−3^ Electrocatalytic water splitting is an environmentally friendly way to produce green hydrogen sustainably by consuming the electricity generated from intermittent renewable energy sources (e.g., wind, hydro, tidal, and solar energy).^3−6^ Hydrogen evolution reaction (HER) is an electrochemical half-reaction of water splitting. It requires high-performance electrocatalysts to proceed expeditiously, particularly under alkaline media where HER is more difficult but oxygen evolution reaction (OER, the other half-cell reaction in water splitting) is much more facile than those in acidic media. It has been recognized that the noble metals exhibit an excellent capability of efficiently catalyzing the HER.^7−9^ However, their high cost and low earth abundance limit their application in large-scale green hydrogen production.^10,11^ Therefore, it is essential to develop low-cost high-performance HER electrocatalysts.

Recently, the earth-abundant and inexpensive transition metals and their carbides, nitrides, phosphides, and chalcogenides have attracted considerable attention in the development of HER catalysts.^12−16^ In particular, due to their excellent electronic conductivity and stability, nickel phosphides (Ni_*x*_P_*y*_) have been widely investigated as a potential substitute for noble metal catalysts for HER.^14,17,18^ There have been a variety of phases obtained for nickel phosphides, such as Ni_3_P,^19^ Ni_5_P_2_,^20^ Ni_12_P_5_,^21^ Ni_2_P,^22^ and Ni_5_P_4_.^23^ The nickel phosphides with different Ni/P ratios have exhibited different catalytic properties for HER. For instance, Pan et al. synthesized Ni_12_P_5_, Ni_2_P, and Ni_5_P_4_ by a thermal decomposition approach, and found that their HER activity followed the order of Ni_5_P_4_ > Ni_2_P > Ni_12_P_5_ in acidic conditions.^24^ In fact, Ni_5_P_4_ has drawn great attention since it has been demonstrated to have HER activity comparable to the Pt. As reported by Laursen et al., in 1 M H_2_SO_4_ solution, the Ni_5_P_4_ exhibits a Tafel slope of 33 mV dec^–1^ and an overpotential of 62 mV at 100 mA cm^–2^, which are close to the values given by Pt.^23^ The high activity was also found on the highly ordered Ni_5_P_4_ nanosheets with largely exposed surfaces synthesized by Ledendecker et al.^25^ Besides, Ni_5_P_4_ was even observed to be highly active under alkaline conditions where the HER is in fact more difficult compared to acidic media.^23,25,26^ Moreover, it has been theoretically and experimentally indicated that the catalytic performance of Ni_5_P_4_ is superior to that of other Ni-based compounds, e.g., Ni_3_S_2_ and Ni_3_N.^27^

Motivated by the excellent catalytic performance of Ni_5_P_4_, extensive efforts have been made to explore the origin of its activity for HER. One explanation given by a few researchers is that the Ni_5_P_4_ has a higher positive charge over Ni atoms and a stronger ensemble effect of P atoms, which leads to an increased binding to the first H atom, and in turn enhances the affinity to the second proton.^24,28^ However, the recent theoretical study has shown that the superior activity of Ni_5_P_4_(0001) is provided by the surface P atoms producing nearly optimal H adsorption, instead of the Ni atoms that bind strongly to the H adsorbates.^29^ In a systematic study of the activity of Ni_5_P_4_ surfaces for HER, Hu et al. confirmed the high activity of P atoms, and revealed a close correlation between the activity and the structural properties on an atomic scale.^30^ However, there is still a lack of in-depth knowledge of the origin of this activity. In particular, there exist limited theoretical studies of the HER activity of Ni_5_P_4_ in alkaline media and the underlying mechanism is still poorly understood. Although it is well-known that the electrocatalytic properties of materials are strongly related to the electronic structures of their surface atoms,^31−33^ in the previous theoretical studies, the electronic nature of Ni_5_P_4_ and its role in the HER activity have not been elucidated fully. Therefore, it is necessary to perform a comprehensive investigation to gain a deeper fundamental understanding of the inherent reasons underlying the high activity of Ni_5_P_4_ for HER.

Herein, a comprehensive study has been conducted to understand the origin of the Ni_5_P_4_ activity for HER through density functional theory (DFT) calculations. The low index (0001) facet of Ni_5_P_4_ was chosen for the activity investigation because it has been experimentally demonstrated to be uniformly exposed.^29,34^ The HER activity of Ni_5_P_4_(0001) was systematically evaluated, and the results show that the surface P atoms act as the active sites rather than Ni. This is in fact in good agreement with the previous finding. Through the transition state analysis, the reaction mechanism of HER on Ni_5_P_4_(0001) was determined and the performance of Ni_5_P_4_(0001) toward water dissociation was also explored. Furthermore, the role of the electronic structure of Ni_5_P_4_(0001) in the HER activity has been interpreted.

## 2 Computational Methods

The Vienna Ab initio Simulation Package (VASP) code based on density functional theory (DFT) was employed to perform the first-principles calculations.^35,36^ The exchange-correlation energy was calculated by the generalized gradient approximation (GGA) of Perdew–Burke–Ernzerhof (PBE).^37^ The interaction between electrons and ions (nucleus) was depicted with the projector augmented wave (PAW) method.^38^ The van der Waals (vdW) interactions were described through the DFT-D3 method.^39^ A high cutoff kinetic energy of 500 eV was applied for the plane-wave basis set to approximate the valence electron densities. The convergence criteria for energy and force were set to 10^–5^ eV and 0.02 eV/Å, respectively. A (√3 × √3) R30° supercell of Ni_5_P_4_(0001) consisting of 108 atoms was constructed to explore the HER process on the surface. The vacuum layer thickness was set to 20 Å in the *z*-direction to eliminate the vertically periodic interaction. The slab dipole correction was applied to avoid the electrostatic effects along the *z*-direction. During structure relaxation, the atoms in the bottom Ni–P layers were frozen, and the number of frozen atoms varied with different surface terminations (Table S1). According to the Monkhorst–Pack scheme, the Brillouin zone was sampled by Γ-centered k-point meshes of 9 × 9 × 3 and 3 × 3 × 1 for the geometry optimization of the unit cell and supercells, respectively, while in the electronic structure calculations for them denser k-point meshes of 12 × 12 × 5 and 5 × 5 × 1 were used, respectively. The climbing image nudged elastic-band method (CI-NEB) was employed to search transition states.^40,41^ The vibrational frequency analysis was subsequently performed to confirm that only one imaginary frequency existed for the structures of the obtained transition states.

Due to the surface slabs of Ni_5_P_4_(0001) being asymmetric, the surface energy (γ) was calculated by the following expression:^42^
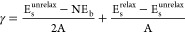
1where the E_s_^unrelax^ and E_s_^relax^ are the total energy of the unrelaxed and relaxed surface slabs, respectively; E_b_ is the total energy of a unit cell of bulk Ni_5_P_4_; N is the number of the unit cells contained in the surface slab; and A is the surface area of the slab model. The first term represents the energy consumed to cleave the bulk Ni_5_P_4_ into two surface slabs, and the second term is the energy generated from the surface relaxation. The surface energy is the sum of the cleavage energy and the relaxation energy.^42^

The Gibbs free energy for H adsorption (Δ*G*_H_) is an excellent descriptor to evaluate the HER performance of one catalyst.^43^ It can be obtained through the equation below:
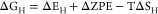
2where Δ*E*_H_ is the hydrogen adsorption energy, ΔZPE represents the zero-point energy correction of H adsorption, T is the temperature of 298 K, and Δ*S*_H_ is the loss of entropy due to the adsorption of the hydrogen atoms on the catalyst surface. The Δ*S*_H_ is approximated as Δ*S*_H_ ≈ −1/2(S_H2_^0^), where the S_H2_^0^ is the entropy of H_2_ in gas phase at standard conditions (298 K, 1 bar). The Δ*E*_H_ can be calculated by the following equation:
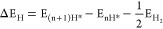
3where E_(n+1)H*_ and E_nH*_ represent the total energy of the surface slabs with n+1 and n adsorbed H atoms, respectively; E_H_2__ is the energy of a hydrogen molecule in gas phase. The ΔZPE can be determined from
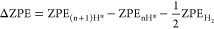
4where ZPE_(n+1)H*_ and ZPE_nH*_ are the zero-point energy of the surfaces adsorbed by n+1 and n H atoms, respectively; and ZPE_H_2__ is the zero-point energy of H_2_ in gas phase. The zero-point energy can be calculated from vibrational frequency calculations as follows:
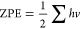
5where *h* is the Planck constant and *v* is the calculated vibrational frequency of H adsorbates.

For transition state analysis, the activation energy (*E*_a_) was computed from
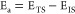
6where E_TS_ and E_IS_ represent the total energy of the transition state and initial state, respectively. In the electronic structure analysis, the net charge (Q_net_) carried by an atom is defined as
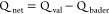
7where Q_bader_ and Q_val_ are the calculated Bader charge and the number of valence electrons that the DFT calculations assume for the atom, respectively. A positive or negative value of the net charge represents that the atom loses or gains electrons (positively and negatively charged). The charge density difference (Δρ) for the H adsorption is defined as Δρ = ρ_H+surf._ – ρ_surf._ – ρ_H_, where ρ_H+surf._, ρ_surf._, and ρ_H_ are the charge densities of the H-adsorbed surface, the clean surface, and the H atom, respectively.

## 3 Results and Discussion

### 3.1 Structure and Stability of Ni_5_P_4_(0001) Surfaces

**Figure 1:**
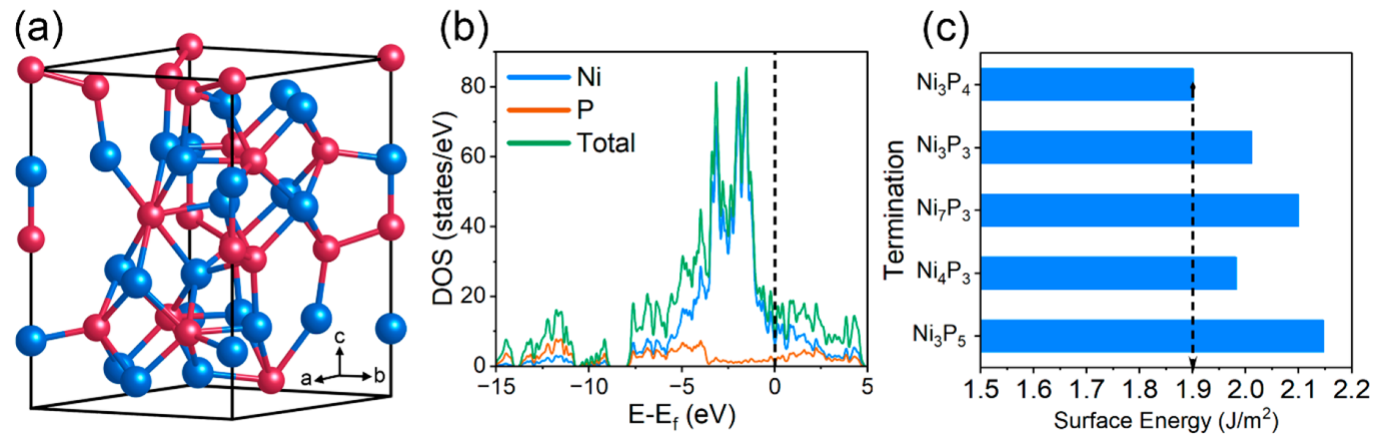
(a) Crystal structure of a Ni_5_P_4_ unit cell. The blue and red spheres represent Ni and P atoms, respectively. (b) Calculated density of states (DOS) of bulk Ni_5_P_4_. (c) Calculated surface energy of five possible surface terminations in the [0001] direction.

Figure 1a shows the structure of Ni_5_P_4_ unit cell, which consists of 36 atoms and has a hexagonal structure with a space group of *P*6_3_*mc*. The optimized lattice constants are *a* = *b* = 6.729 Å and *c* = 10.890 Å, which are in good agreement with the reported experimental and computational results, as summarized in Table S2. Figure 1b shows the calculated density of states (DOS) of the bulk Ni_5_P_4_. It can be observed that there are electronic states across the Fermi level, which are mainly contributed by the Ni atoms. This demonstrates that Ni_5_P_4_ exhibits a metallic feature, facilitating electron transfer during electrocatalytic HER processes.

Analysis of surface stability was carried out to determine the most stable surface termination of Ni_5_P_4_ in the [0001] direction. As presented in Figure 1a, the unit cell of Ni_5_P_4_ is composed of two identical halves along the [0001] direction, one of which is rotated 180° around the (0001) axis with respect to the other one. In half a unit cell, the atoms have a complex arrangement in the [0001] direction and the delineation of atomic layers is less clear. By removing the outermost atoms or atomic layers from the surface in the [0001] direction, as illustrated in Figure S1, five possible surface terminations were obtained. According to the composition of the exposed surface atoms, they are referred to as Ni_3_P_4_, Ni_3_P_3_, Ni_7_P_3_, Ni_4_P_3_, and Ni_3_P_5_ surface terminations, respectively. Figure 1c shows a comparison of the calculated surface energy of the Ni_5_P_4_(0001) with different terminations. It is clearly demonstrated that the Ni_3_P_4_-terminated Ni_5_P_4_(0001) has the lowest surface energy, which implies that Ni_3_P_4_ is the most stable termination. By energetics consideration, it is predicted that the Ni_3_P_4_ termination has a high probability of being exposed to the reactant during HER catalysis.^44^ Therefore, the Ni_3_P_4_ termination was selected to investigate the catalytic activity of Ni_5_P_4_(0001) for HER.

### 3.2 HER Activity of Ni_5_P_4_(0001) with Ni_3_P_4_ Termination

**Figure 2:**
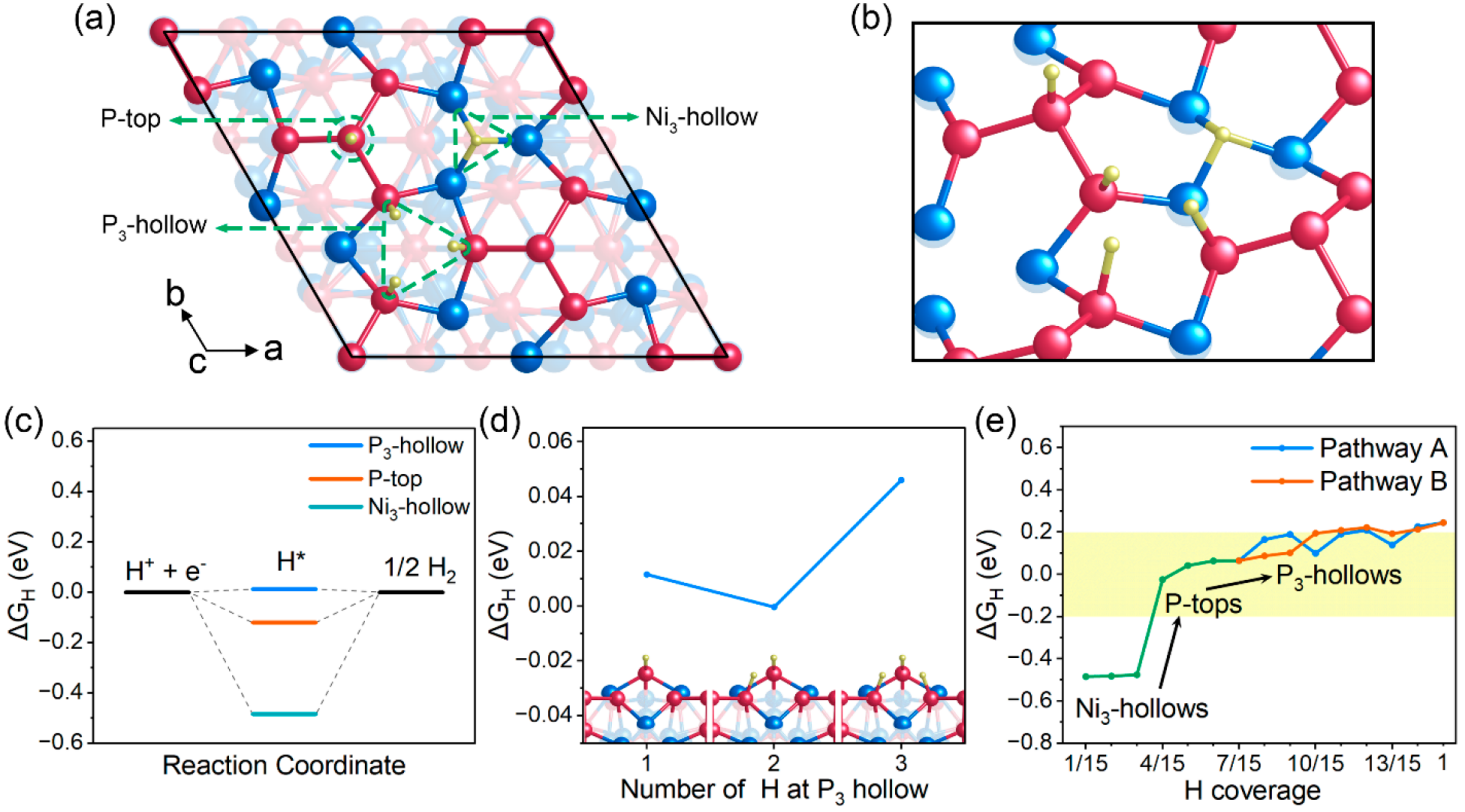
(a and b) Stable geometries of H adsorption at three kinds of sites on the Ni_3_P_4_-terminated Ni_5_P_4_(0001) surface. Note that a P_3_ hollow can adsorb three H atoms, each of which bonds to one P atom of the P_3_ hollow, while a Ni_3_ hollow can only stabilize one H atom, bonding to the three Ni atoms, over the hollow center. The blue, red, and yellow spheres represent Ni, P, and H atoms, respectively. (c) Three-state free energy diagram for HER at the different surface sites. (d) Gibbs free energy of H adsorption as a function of the number of adsorbed H atoms at the P_3_-hollow site. (e) Gibbs free energy of H adsorption (Δ*G*_H_) as a function of H coverage. The yellow region refers to the preferable range of Δ*G*_H_ for HER (i.e., |Δ*G*_H_ | < 0.2 eV).

As shown in Figure 2a, the Ni_3_P_4_-terminated Ni_5_P_4_(0001) surface consists of repeating triangular Ni_3_ connected with each other by tetrahedral P_4_. The P corners of three adjacent tetrahedral P_4_ gather around a common point, creating a P_3_ hollow. We have considered all possible sites on the Ni_3_P_4_ termination to identify stable hydrogen adsorption sites. After full structure relaxation, the H adsorption was finally stabilized at three types of sites, i.e., Ni_3_-hollow sites, P-top sites (the top of central P in tetrahedral P_4_), and P_3_-hollow sites. The optimized geometry of H adsorption for each kind of site is schematically demonstrated in Figure 2a and 2b. It has been found that a P_3_-hollow site can accommodate three H atoms, each of which bonds to one P atom there, while a Ni_3_-hollow site can stabilize only one H atom, bonding to the three Ni atoms, over their center. According to the calculated H adsorption energy (Δ*E*) listed in Table S3, the Ni_3_-hollow sites are identified as the preferable sites for H adsorption with the largest negative Δ*E* value of −0.732 eV, whereas the P_3_-hollow sites and P-top sites exhibit relatively weaker adsorption toward H atoms than the Ni_3_-hollow sites, with much smaller negative Δ*E* value of −0.276 eV and −0.422 eV, respectively.

The Gibbs free energy of H adsorption (Δ*G*_H_) has been generally perceived as a suitable descriptor for evaluating HER performance. The Δ*G*_H_ should be close to zero on excellent HER electrocatalysts, which indicates an ideal trade-off between adsorption and release of H atoms during surface electrocatalysis. Either too weak or too strong binding of H atoms to the catalyst surface can lead to an inefficient HER process. Figure 2c and Table S3 show the calculated Δ*G*_H_ for the Ni_3_P_4_-terminated Ni_5_P_4_(0001) surface. The P_3_-hollow sites have a nearly thermoneutral H-adsorption with a Δ*G*_H_ value of 0.012 eV that is close-to-zero, signifying that the P_3_-hollow sites can deliver near-optimal HER catalytic activity. The P-top sites are also highly active for HER (Δ*G*_H_ = −0.121 eV), but their activity is lower than P_3_-hollow sites. The biggest negative Δ*G*_H_ (−0.484 eV) was found for Ni_3_-hollow sites. With such a large negative Δ*G*_H_, the H atoms can be readily adsorbed at the Ni_3_-hollow sites, but the release of H atoms from these sites is very difficult, leading to an inefficient HER process. In addition, given that each P_3_-hollow site can adsorb two additional H atoms, we investigated the effect of the number of adsorbed H atoms at one P_3_-hollow site on its HER activity. As shown in Figure 2d, the adsorption of the second H atom becomes easier with a more negative Δ*G*_H_. However, when one more (i.e., the third) H atom is adsorbed at the site, the Δ*G*_H_ is increased to 0.046 eV, more positive than that of the adsorption of only one H atom. This indicates that the activity of the P_3_-hollow site is diminished due to the interactions among adsorbed H atoms when the site is fully occupied by three H atoms. Despite that, this Δ*G*_H_ value of 0.046 eV remains close to zero, demonstrating that the high catalytic activity of the P_3_-hollow site for HER can be maintained with the increasing number of the adsorbed H atoms at the site.

Furthermore, we explored the dependence of the HER activity of Ni_3_P_4_-terminated Ni_5_P_4_(0001) surface on H coverage. The H coverage is defined as the ratio of the number of adsorbed H atoms to the number of surface adsorption sites. Since the above results suggest that the interaction of the adsorbed H atoms at the P_3_-hollow sites can affect the Δ*G*_H_, we considered two pathways (A and B) for increasing the H coverage at the P_3_-hollow sites, as schematically illustrated in Figure S2. In pathway A, the H atoms are adsorbed at one P_3_-hollow site until the latter is fully occupied, then another P_3_-hollow site begins to adsorb H atoms, while in pathway B, the adsorption of the next H atom always occurs at another P_3_-hollow site. At a same H coverage (at least two H atoms are adsorbed at the same P_3_-hollow site), there are different interactions of H atoms at P_3_-hollow sites in the pathways A and B. Figure 2e and Table S4 show the calculated Δ*G*_H_ values under various H coverages in the pathways A and B. The yellow region in Figure 2e refers to the preferable range of Gibbs free energy of H adsorption (|Δ*G*_H_ | < 0.2 eV).^45^ Clearly, pathway A has greater Δ*G*_H_ values than pathway B between 7/15 and 9/15 H-coverage due to the stronger interaction of adsorbed H atoms at the P_3_-hollows in pathway A. Subsequently, at 10/15 H-coverage, the Δ*G*_H_ value of pathway A is greatly reduced to a value lower than that of pathway B. This is because, for 10/15 H-coverage, the H-adsorption via pathway A occurs at the nonoccupied P_3_-hollow site, while in pathway B the P_3_-hollow site is already occupied by one H atom. This leads to stronger interaction between the adsorbed H atoms in pathway B than in A, weakening the H adsorption. Between 10/15 and 13/15 H-coverage, pathway A has greater Δ*G*_H_ value at P_3_-hollow sites than pathway B. It is noted that the Δ*G*_H_ values are increased to over 0.2 eV at 12/15 H-coverage for pathway A (Δ*G*_H_ = 0.209 eV) and 11/15 H-coverage for pathway B (Δ*G*_H_ = 0.209 eV), respectively, but at a similar coverage of 13/15, they are reduced back to below 0.2 eV. When H-coverage is over 14/15, the HER activity becomes low with the Δ*G*_H_ being greater than 0.2 eV. Therefore, it is evidenced that the HER is more facile over a wider range of H coverages via pathway A than B, i.e., the H-coverage of 4/15 to 11/15 for pathway A and of 4/15 to 10/15 for pathway B. Overall, the Ni_3_P_4_-terminated Ni_5_P_4_(0001) surface can maintain a high activity for HER over a large H-coverage range.

### 3.3 Mechanism of HER on Ni_5_P_4_(0001)

The HER process can proceed through two distinct mechanisms, the Volmer-Heyrovsky mechanism and the Volmer–Tafel mechanism. Both mechanisms start with the Volmer step producing the key reactive intermediate H* (* represents the hydrogen adsorption site), followed by the production of H_2_ through either the Heyrovsky step or Tafel step. Under acidic conditions, these three steps can be expressed as below:
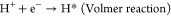

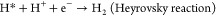

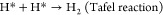
On the Ni_3_P_4_-terminated Ni_5_P_4_(0001) surface, the HER can proceed very fast through the Volmer–Heyrovsky pathway at the P_3_-hollow sites since their Δ*G*_H_ values are nearly zero.^46^ The Volmer–Heyrovsky pathway is also viable at P-top sites, but their HER rates are relatively lower than the P_3_-hollow sites since the P-top sites have a less favorable Heyrovsky step. Regarding the Ni_3_-hollow sites, their strong binding to the H atoms favors the Volmer step but in the meantime makes it difficult to release the adsorbed H atom to produce H_2_, hindering the Heyrovsky step.

**Figure 3:**
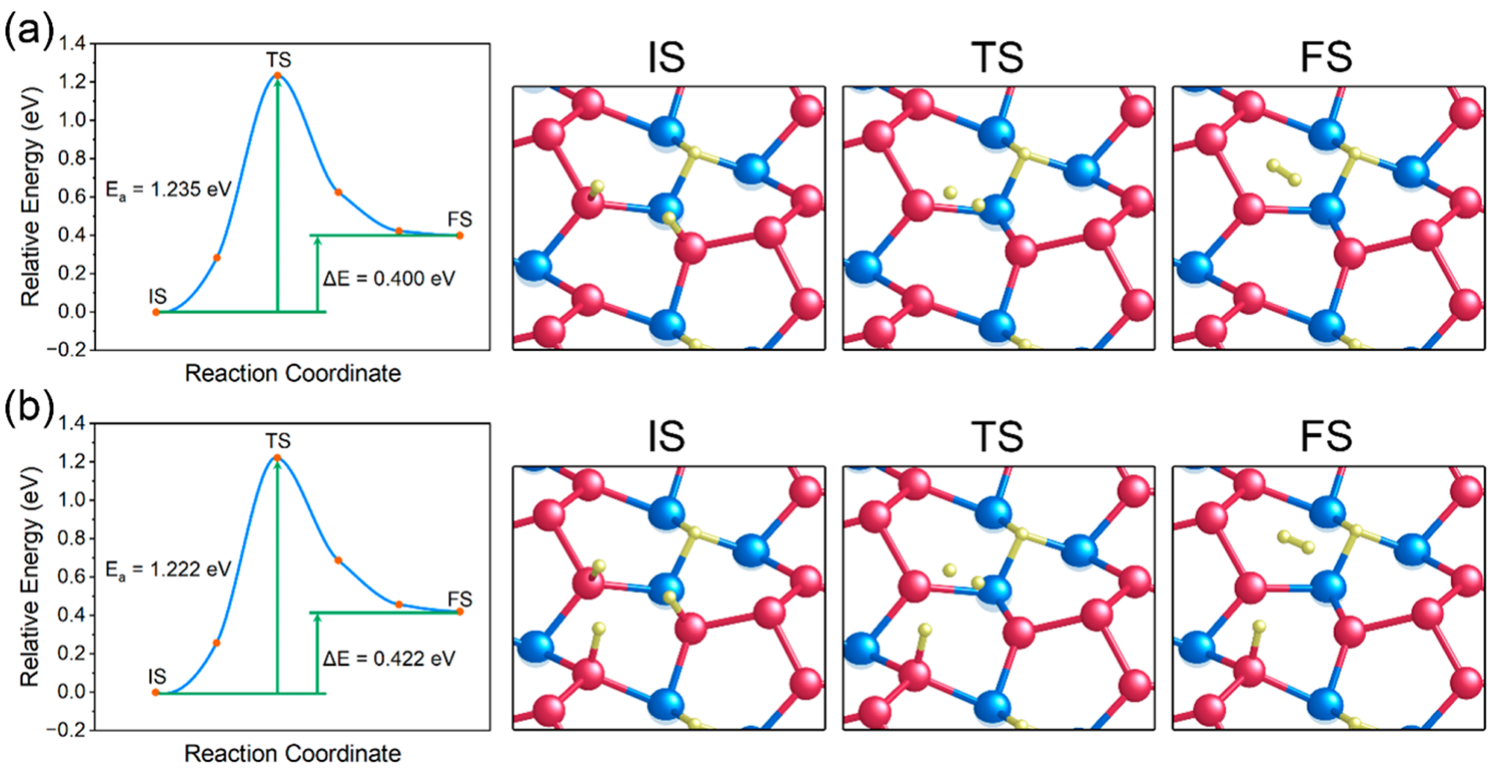
Reaction profiles of the Tafel step at the P_3_-hollow sites with (a) two and (b) three adsorbed H atoms at each site. Also shown in the figure (right side) are the corresponding surface structures of the initial states (IS), transition states (TS) and final states (FS) in (a) and (b).

The Tafel step is a process in which two adsorbed H atoms combine to produce a H_2_ molecule. One of the reasons for the excellent HER performance of Pt catalysts is that they have a fast Tafel step;^47^ thus, we explored the viability of the Tafel step on the Ni_3_P_4_-terminated Ni_5_P_4_(0001) surface. Due to the P_3_-hollow sites being the main active sites for HER, we calculated the energy barriers of the Tafel reactions at the P_3_-hollow sites. As shown in Figure 3, we considered two kinds of H adsorption configurations at P_3_-hollow sites as the beginning of the Tafel reaction. One is the P_3_-hollow site partially occupied by two H atoms, while the other is fully occupied by three H atoms, two of which subsequently combine to form H_2_. However, both cases were found to possess high energy barriers of 1.235 and 1.222 eV, suggesting the sluggish kinetics of H_2_ generation at the P_3_-hollow sites through Tafel reactions. A previous study by Ling et al.^48^ has shown that a greatly reduced H–H distance between two neighboring adsorbed H atoms can lead to a low energy barrier for the Tafel reaction. In their study, a very low energy barrier of 0.48 eV for the Tafel reaction was obtained at those active sites where the two adsorbed H atoms are about 1.5 Å apart, while, in our work, the H–H distances are found to be much greater, 2.20 and 2.19 Å for both H adsorption configurations, respectively. Therefore, we believe that the long H–H distance between two adjacent adsorbed H atoms at the P_3_-hollow sites is a crucial factor causing their high kinetic barriers for Tafel reactions. Overall, it can be concluded that in acidic environments the energetically favorable Volmer–Heyrovsky pathway is the dominant mechanism for the HER on the Ni_3_P_4_-terminated Ni_5_P_4_(0001) surface. This is in agreement with the experimental result that a Volmer–Heyrovsky mechanism for Ni_5_P_4_ was indicated by the obtained Tafel slope of 40 mV dec^–1^.^25^

**Figure 4:**
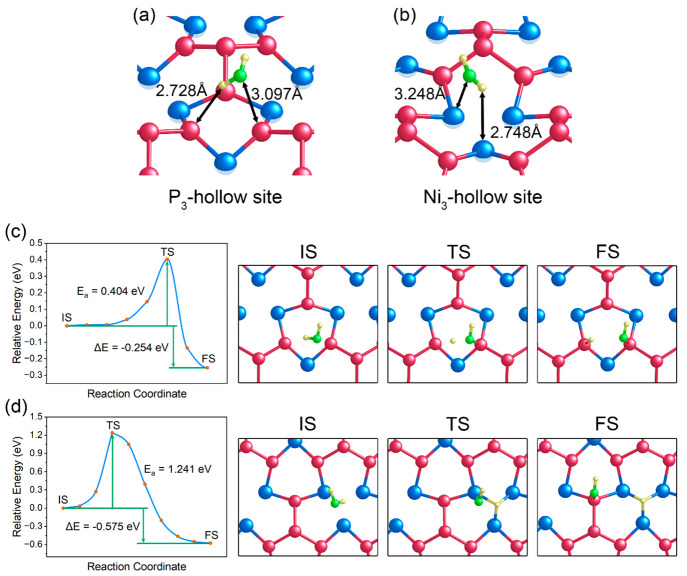
Stable configurations of water adsorption at the (a) P_3_-hollow site and (b) Ni_3_-hollow site. The blue, red, yellow, and green spheres represent Ni, P, H, and O atoms, respectively. Reaction profiles of H_2_O dissociation at the (c) P_3_-hollow site and (d) Ni_3_-hollow site; also shown in the figure (right side) are the corresponding top views of the initial states (IS), transition states (TS), and final states (FS) of water dissociation in (c) and (d).

Due to the scarcity of protons in alkaline electrolytes, water dissociation, also known as the alkaline Volmer step (H_2_O + e^–^ → H* + OH^–^), becomes the main source providing H intermediates. Therefore, the kinetic barrier for water dissociation was examined for the Ni_3_P_4_-terminated Ni_5_P_4_(0001) surface. It was found that on the surface there were two kinds of sites for stable adsorption of molecular water, i.e., P_3_-hollow and Ni_3_-hollow sites. Figure 4a and 4b show the stable geometries of the water molecule adsorbed at P_3_-hollow and Ni_3_-hollow sites, respectively. The adsorption energy was calculated to be −0.183 eV and −0.289 eV on the P_3_-hollow and Ni_3_-hollow sites, respectively, suggesting that the Ni_3_-hollow sites have a stronger adsorption for H_2_O than the P_3_-hollow sites. At the P_3_-hollow site, the H_2_O molecule is stabilized by one P atom with a P–O bond length of 3.097 Å. Meanwhile, another P atom at the site tends to attract the H atom of H_2_O, giving a shorter P–H bond length of 2.728 Å. For the H_2_O adsorption at the Ni_3_-hollow site, the H atom of H_2_O is attracted to the hollow center with an average Ni–H bond length of 2.748 Å, whereas the O atom of H_2_O is at a distance of 3.248 Å from the nearest Ni atom. This indicates that the Ni_3_-hollow site has a greater binding affinity for the H atoms of H_2_O than the O atom during water adsorption. Besides, as shown in Table S5, in comparison to the free molecular water in vacuum (0.972 Å), the O–H bonds of the adsorbed H_2_O were observed to be slightly elongated, i.e., 0.977 and 0.981 Å for P_3_-hollow and Ni_3_-hollow sites, respectively. This suggests that the O–H bonds in the water molecule were weakened. In addition, the longer O–H bond for the water at Ni_3_-hollow sites than that at the P_3_-hollow sites indicates a stronger activation of the O–H bonds over the Ni_3_-hollow sites.

The transition state analysis was then performed to determine the energy barriers for water dissociation at P_3_-hollow and Ni_3_-hollow sites. Figure 4c and 4d show the energy changes as a function of the reaction coordinate, together with the schematic representations of the initial state (IS), transition state (TS), and final state (FS) of water dissociation at P_3_-hollow and Ni_3_-hollow sites, respectively. It can be observed that the water dissociation at both sites is exothermic, indicating a thermodynamically favorable process. In addition, the energy barrier for water dissociation at Ni_3_-hollow sites (1.241 eV) is much higher than at P_3_-hollow sites (0.404 eV). This is because at the P_3_-hollow site, the H and OH species generated from water dissociation can be directly captured by the P_3_-hollow site at once. However, since the Ni_3_-hollow site can only adsorb one H atom, the generated OH specie needs to diffuse to the P_3_-hollow site to be stabilized. Although the Ni_3_-hollow site can activate the O–H bond in H_2_O more favorably during H_2_O adsorption, the diffusion of the OH species on the surface inevitably introduces an extra kinetic barrier, resulting in an increase in the energy barrier for H_2_O dissociation at the Ni_3_-hollow site. Therefore, the H adsorbate can be preferentially generated at P_3_-hollow sites during water dissociation, favoring the alkaline HER. Besides, the energy barrier for water dissociation on Ni_5_P_4_(0001) (0.404 eV) is much lower in comparison to that on Ni_2_P (0.82 eV) reported by Cross et al.^49^ This is in accordance with the experimental finding that Ni_5_P_4_ exhibits a higher catalytic activity for alkaline HER than Ni_2_P.^23^

### 3.4 Origin of Ni_5_P_4_(0001) Activity for HER

**Figure 5:**
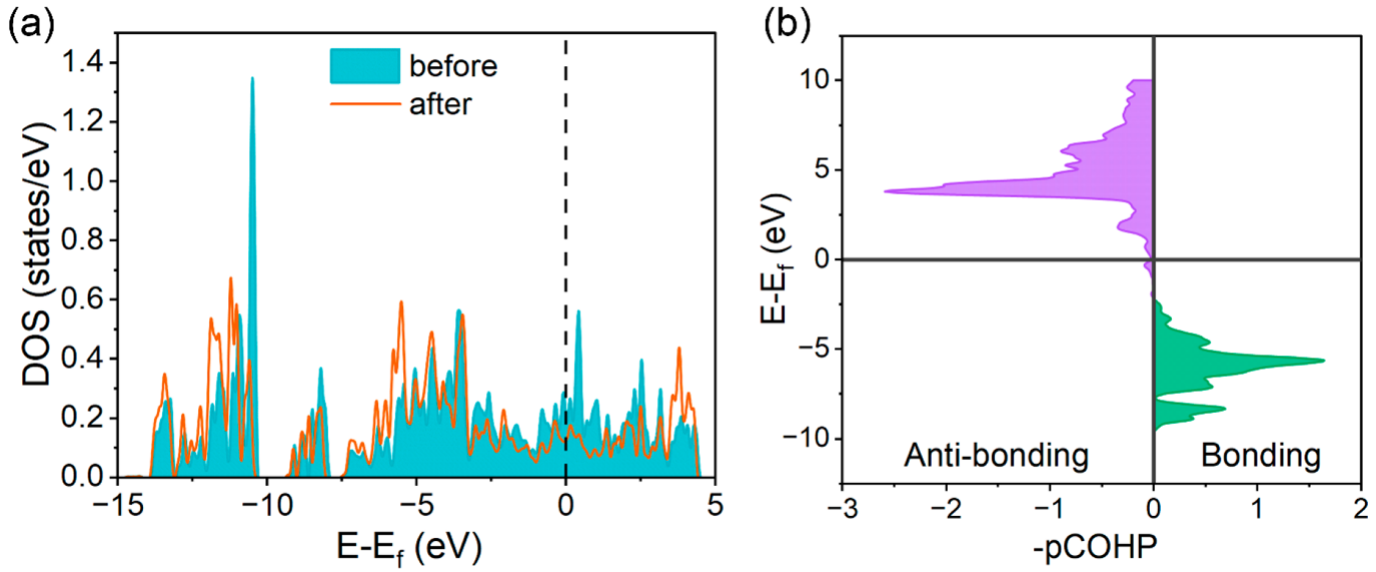
(a) Calculated density of states (DOS) for the P_3_-hollow site before and after H adsorption. The zero energy is set to the Fermi level (dash line). (b) Calculated projected crystal orbital Hamilton population (pCOHP) for the P–H bond at the P_3_-hollow site.

The above results have indicated that the P_3_-hollow sites of the Ni_3_P_4_-terminated Ni_5_P_4_(0001) surface exhibit a superior activity for HER. To gain insight into the origin of the activity of P_3_-hollow sites, a series of electronic structure analyses were performed. We first investigated the bonding characteristics of the P_3_-hollow site with H adsorbates. Figure 5a depicted the calculated density of states (DOS) of the P_3_-hollow site before and after H adsorption. It can be seen that after H adsorption the total DOS is reduced around the Fermi level. Besides, the total DOS below the Fermi level shifts downward to lower energy, while a localized peak is formed at a higher energy above the Fermi level. These changes can be explained by the underlying mechanism of bond formation. When the H atom approaches the P_3_-hollow site on the surface, the coupling of electronic states between them leads to the hybridized energy levels, shifting downward to form bonding states and shifting upward to form antibonding states. The bonding states are well below the Fermi Level and thus fully filled, while the antibonding states are usually located across the Fermi Level and thus partially filled. The adsorption strength is strongly associated with the filling of antibonding states. To intuitively demonstrate the bonding and antibonding states for the P–H bond, the projected crystal orbital Hamilton population (pCOHP) analysis was performed. As shown in Figure 5b, the bonding states of the P–H bond are positioned well below the Fermi level and are thus fully filled. Most of antibonding states are above the Fermi level, while only a small fraction of antibonding states is below the Fermi level, indicating that few antibonding states are filled. This favorable interaction of electronic states leads to the P_3_-hollow site producing stable adsorption toward H atoms during the HER process.

**Figure 6:**
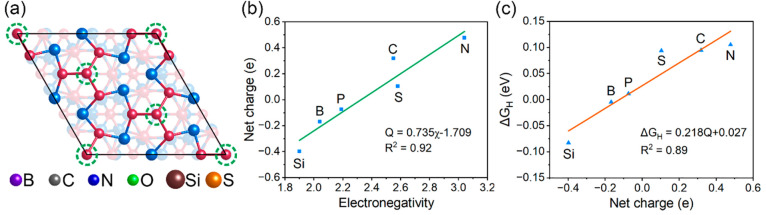
(a) Schematic illustration of the strategy of employing substitutional doping of nonmetal atoms to adjust the net charge of the P_3_-hollow sites. The green dash circle indicates the P atoms to be replaced by the six nonmetal atoms listed there. (b) Linear correlation between the net charge carried by the P_3_-hollow sites and the electronegativity of the central doped atoms. (c) Linear correlation between the Gibbs free energy for H adsorption at P_3_-hollow sites on the doped surfaces and the net charge carried by the sites.

Next, we investigated the effect of the net charge (defined by eq 7) carried by the P_3_-hollow site on its HER activity by means of Bader charge analysis. To adjust the net charge of the P_3_-hollow site, the strategy of exploring substitutional doping by nonmetal atoms was employed here. As illustrated in Figure 6a, the central P atoms of the tetrahedral P_4_ on the Ni_3_P_4_ termination were substituted by various nonmetal atoms, i.e., B, C, N, O, Si, and S. The electronegativity of these nonmetal atoms follows the order of O > N > S > C > P > B > Si. Figure S3 presents the optimized geometries of the doped Ni_3_P_4_-terminated Ni_5_P_4_(0001) surfaces. It can be seen that the structures of these doped surfaces remain consistent with the pristine surface, except for the O-doped surface, where a significant structural distortion occurs due to the strong electronegativity of O atoms. Therefore, doping of O atoms is not considered in the following analysis. As observed in Figure 6b, the net charge of the P_3_-hollow site presents a clear linear relationship with the electronegativity of the central atoms. This indicates that the electronegativity difference between the doped atom and P successfully evokes the charge redistribution and modulates the net charge of the P_3_-hollow site. Specifically, the dopant atoms (O, N, S, and C) with a higher electronegativity than P make their surrounding P atoms at P_3_-hollow sites more positively charged as they attract the electrons from the latter, and vice versa. The adjusted net charge ranges from −0.398 to 0.478 e. The highest and lowest net charges are given by the doping of Si and N atoms, respectively. The Δ*G*_H_ of P_3_-hollow sites on the doped surface was calculated to assess their HER activity. As shown in Figure 6c, the doping of Si atoms gives the most negative Δ*G*_H_ of −0.083 eV at P_3_-hollow sites while the most positive Δ*G*_H_ (0.105 eV) is found for the N doping. This shows that the Δ*G*_H_ values at the P_3_-hollow sites on the doped surfaces remain within the preferable Gibbs free energy range (|Δ*G*_H_| < 0.2 eV) for the HER, as already mentioned above. Besides, the Δ*G*_H_ is linearly correlated with the net charge with a reasonable fitting factor *R*^2^ of 0.89. A larger negative net charge is observed to generate a more negative value of Δ*G*_H_, which implies that the P_3_-hollow sites with more electrons can provide stronger adsorption toward H atoms. Importantly, it can be seen that the P_3_-hollow sites on the pristine surface have an optimal net charge that leads to the nearly thermoneutral H adsorption. This is responsible for the superb activity of the pristine Ni_3_P_4_-terminated Ni_5_P_4_(0001) surface for HER.

**Figure 7:**
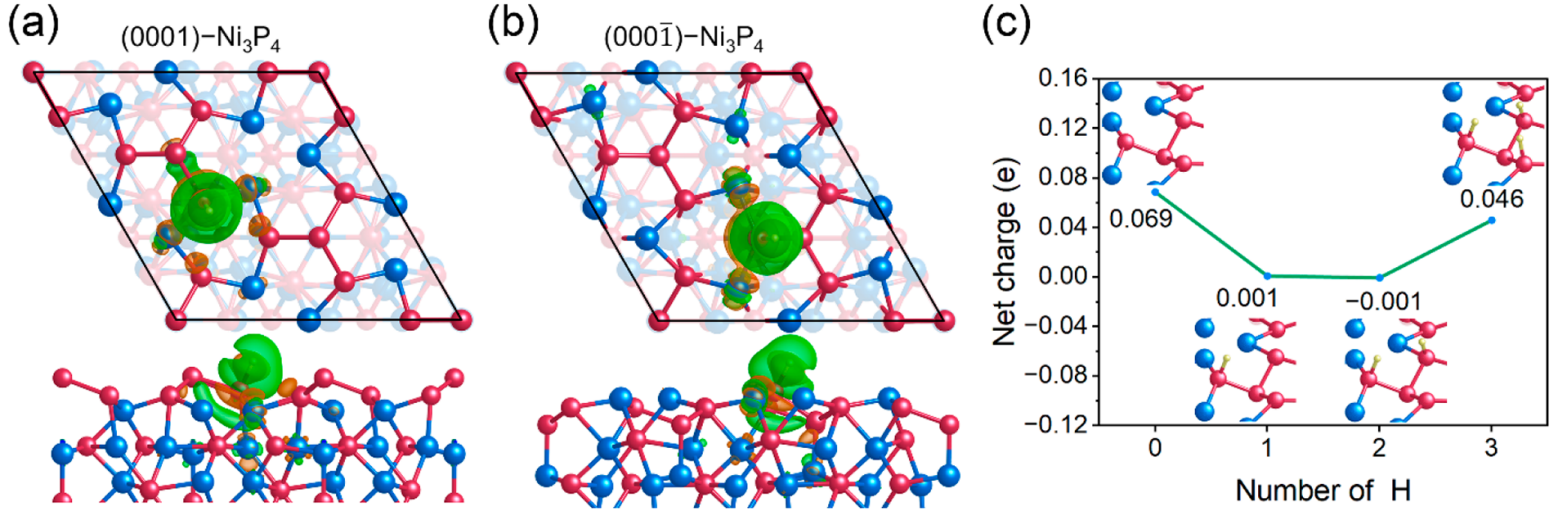
Top and side views of the charge density difference of (a) the (0001)-Ni_3_P_4_ and (b) the (0001̅)-Ni_3_P_4_ surfaces before and after H adsorption. Orange and green regions denote charge accumulation and depletion, respectively. The iso-surface value was set to 0.0015 e/Bohr^3^. (c) Net charge on the central P atom as a function of the number of the H atoms adsorbed on the neighboring P_3_-hollow sites on the (0001̅)-Ni_3_P_4_ surface.

Furthermore, charge transfer analysis was also performed to understand the adsorbate–surface interaction. Figure 7a shows the charge density difference of the Ni_3_P_4_-terminated Ni_5_P_4_(0001) surface before and after H adsorption. It can be observed that the H adsorption at the P_3_-hollow site triggers a significant charge transfer; i.e., there is noticeable electron depletion (green) around the P_3_-hollow site and the H atom, while electron accumulation in the middle between the two atoms. This indicates the strong interaction between the two atoms and the formation of the P–H bond during H adsorption, in agreement with the DOS and pCOHP analyses described above. Another region of electron depletion is found under the P_3_-hollow site, and the electrons move toward the P_3_-hollow site. The direction of this charge transfer can be attributed to the P_3_-hollow site gaining electrons from the surroundings to balance its reduced charge during the formation of the P–H bond. Besides, an obvious charge transfer between the P_3_-hollow site and the surrounding Ni atoms also occurs. This corresponds to the observed increase in the lengths of the Ni–P bonds after H adsorption (Figure S4), which indicates the weakened strength of neighboring Ni–P bonds.

To further understand the effect of charge transfer on the HER activity of P_3_-hollow sites, we now discuss the Ni_3_P_4_-terminated Ni_5_P_4_(0001̅) surface since we found that the activity of P_3_-hollow sites on this surface suffers from poor charge transfer. It is worth noting that although the Ni_5_P_4_(0001) and Ni_5_P_4_(0001̅) surfaces have the same termination of Ni_3_P_4_ composition, they are still structurally distinct, due to the bulk Ni_5_P_4_ lacks mirror symmetry along the [0001] direction, as illustrated in Figure S5. We refer to the two surfaces as (0001)-Ni_3_P_4_ and (0001̅)-Ni_3_P_4_ in the following discussion, respectively. The net charge of the P_3_-hollow site on the (0001̅)-Ni_3_P_4_ is calculated to be −0.151 e, more negative than that on the (0001)-Ni_3_P_4_ (−0.075 e). However, the P_3_-hollow site on the (0001̅)-Ni_3_P_4_ (Δ*G*_H_ = 0.317 eV) exhibits a much weaker ability toward H adsorption than the (0001)-Ni_3_P_4_ (Δ*G*_H_ = 0.012 eV), which is apparently inconsistent with the above result that the higher net charge of the P_3_-hollow site can generate stronger H adsorption. After examining the charge distribution on the surface, we found that the central P atom of the tetrahedral P_4_ on the (0001)-Ni_3_P_4_ is positively charged with a net charge of 0.069 e. This leads to the central P atom, as a nonmetal element, having stronger attraction to electrons. For instance, Figure 7b shows a significant charge transfer occurs between the P_3_-hollow site with the neighboring atoms after H adsorption. The Bader analysis (Figure 7c) indicates that after the first H atom is adsorbed at the P_3_-hollow site, the net charge of the central P atom increases by 0.068 e. Clearly, this unfavorable charge transfer prevents the P_3_-hollow site from gaining enough electrons to form a stronger P–H bond. On the other hand, the adsorption of the first H atom makes the central P atom less positively charged (0.001 e), weakening the attraction of the central P atom toward electrons. Thus, upon the adsorption of the second H atom, the central P atom attracts fewer electrons (Figure 7c); i.e., the net charge of the central P atom decreases from 0.001 to −0.001 e, lowering the Δ*G*_H_ to 0.244 eV and favoring the H adsorption at the P_3_-hollow site. With the central P atom turning to be negatively charged, the central P atom does not attract electrons from the surrounding atoms but acts as an electron donor to provide the electrons for the P_3_-hollow site to bind to the H adsorbate. This leads to Δ*G*_H_ being further reduced to 0.142 eV, signifying the much easier adsorption of the third H atom. Compared with the (0001̅)-Ni_3_P_4_ surface, the (0001)-Ni_3_P_4_ surface has a favorable charge distribution, benefiting the charge transfer. For example, the central P atom is negatively charged with the net charge of −0.012 e on the clean (0001)-Ni_3_P_4_ surface. After the adsorption of one H atom, the net charge is increased to −0.005 e and the central P atom remain negatively charged. This demonstrates that the central P atom can remain as an electron donor to the P_3_-hollow site to promote the H adsorption. Therefore, the (0001)-Ni_3_P_4_ surface has a favorable charge transfer, facilitating the HER process.

## 4 Conclusions

DFT calculations were successfully performed to investigate the origin of the HER activity of Ni_5_P_4_(0001) model catalyst. The results show that the lowest surface energy was obtained on the Ni_5_P_4_(0001) surface exposing Ni_3_P_4_ termination. The P_3_-hollow sites provide thermoneutral hydrogen adsorption, thus contributing to the high HER activity of Ni_5_P_4_(0001). It has also been observed that the surface remains catalytically active over a wide H coverage. The optimal free energy for hydrogen adsorption suggests that HER is facile via the Volmer–Heyrovsky mechanism, while the Tafel reaction is kinetically unfavorable due to its large energy barrier. The P_3_ hollow sites were also found to provide a low energy barrier toward water dissociation, favoring the alkaline HER. A series of electronic structure analyses performed have gained a deeper understanding of the activity origin. The results indicate that the superior HER activity of Ni_5_P_4_(0001) can be attributed to three main factors. First, the DOS and COHP analyses revealed a favorable interaction of electronic states, leading to stable adsorption of P atoms toward H atoms. Second, from the Bader charge analysis, it was found that the strength of H adsorption at P_3_-hollow sites linearly increases with the electrons carried by them. The appropriate net charge at P_3_-hollow sites leads to the optimal close-to-zero Δ*G*_H_. Third, highly efficient electron transfer between the P_3_-hollow sites and their neighboring atoms also contributed to the facile HER process.

## Supporting Information Available

Various numbers of the frozen atoms in the slab models; Lattice parameters of Ni_5_P_4_; Calculated hydrogen adsorption energies and the Gibbs free energy for H adsorption; Calculated geometry and energy of water adsorption; Illustration of generating surface termination; Illustration of two pathways for H coverage increasing; Optimized geometries of doped surfaces; Illustration of the structure difference between the (0001) and (0001̅) surfaces. (PDF)
