# Supplementary material for: Insights into the Origin of High Activity of Ni5P4(0001) for Hydrogen Evolution Reaction
Source: J Phys Chem C Nanomater Interfaces. 2023 Mar 9;127(11):5385–94. doi: 10.1021/acs.jpcc.3c00238 (PMC10041637; doi:10.1021/acs.jpcc.3c00238)
Supplement: Supplementary file 1 — jp3c00238_si_001.pdf [file jp3c00238_si_001.pdf]

Supporting Information

Insights into the Origin of High Activity of Ni_5P_4(0001) for Hydrogen Evolution Reaction

Yang Yang[a], Xiao Lin[b], Yang Li[a], Tian Sheng[c], Shaoan Cheng[d], Xiaoming Sun[e], and Wen-Feng Lin*, [a]

[a] Department of Chemical Engineering, Loughborough University, Loughborough, Leicestershire, LE11 3TU, United Kingdom.

[b] Department of Chemical Engineering and Biotechnology, University of Cambridge, Cambridge, CB3 0AS, United Kingdom.

[c] College of Chemistry and Materials Science, Anhui Normal University, Wuhu, 241000, China.

[d] State Key Laboratory of Clean Energy, Department of Energy Engineering, Zhejiang University, Hangzhou 310027, China.

[e] State Key Laboratory of Chemical Resource Engineering, Beijing Advanced Innovation Center for Soft Matter Science and Engineering, College of Chemistry, Beijing University of Chemical Technology, Beijing, 100029, China.

* Corresponding author

Email address: **w.lin@lboro.ac.uk** (Wen-Feng Lin)

Table S1. Various number of the frozen atoms in the slab models with different terminations employed in the surface calculations. In the surface stability analysis, the (1×1) supercell was used, while for the H adsorption, the supercell was expanded to (√3 × √3).

Slab size	(1×1)					(√3 × √3)	
Termination	Ni_3P_4	Ni_3P_3	Ni_7P_3	Ni_4P_3	Ni_3P_5	Ni_3P_4/ (0001)	Ni_3P_4/ $(000\bar{1})$
Frozen atoms	7Ni, 4P	7Ni, 5P	3Ni, 5P	6Ni, 5P	7Ni, 6P	21Ni, 12P	21Ni, 12P

Table S2. Calculated lattice parameters (Å) of bulk Ni_5P_4 in comparison with the previously reported experimental and theoretical results.

	Lattice parameters (Å)	
	a and b	c
This work	6.729	10.890
Calc.[1]	6.780	10.970
Expt.[2]	6.789	10.989
Expt.[3]	6.792	10.992

Table S3. The Gibbs free energy of H adsorption (ΔG_H) at the P_3-hollow, P-top and Ni_3-hollow sites of the Ni_3P_4-terminated Ni_5P_4(0001) surface. The structures of H adsorption at corresponding sites are shown in Figure 2a.

Adsorption site	ΔE (eV)	ΔZPE (eV)	ΔG_H (eV)
P_3-hollow	-0.276	0.086	0.012
P-top	-0.422	0.098	-0.121
Ni_3-hollow	-0.732	0.045	-0.484

Table S4. The Gibbs free energy of H adsorption (ΔG_H) as a function of the H coverage on the Ni_3P_4-terminated $Ni_5P_4(0001)$ surface along pathways A and B. The corresponding sites for H adsorption along pathways A and B are shown in Figure S2.

Coverage	Pathway	ΔE (eV)	ΔZPE (eV)	ΔG_H (eV)
1/15	-	-0.732	0.045	-0.484
2/15	-	-0.729	0.046	-0.481
3/15	-	-0.724	0.048	-0.474
4/15	-	-0.326	0.099	-0.025
5/15	-	-0.259	0.099	0.042
6/15	-	-0.236	0.097	0.064
7/15	A	-0.217	0.080	0.065
	B	-0.217	0.080	0.065
8/15	A	-0.113	0.076	0.166
	B	-0.194	0.079	0.088
9/15	A	-0.088	0.075	0.189
	B	-0.179	0.080	0.103
10/15	A	-0.183	0.081	0.100
	B	-0.082	0.075	0.195
11/15	A	-0.086	0.075	0.192
	B	-0.069	0.076	0.209
12/15	A	-0.067	0.074	0.209
	B	-0.055	0.075	0.223
13/15	A	-0.142	0.079	0.139
	B	-0.086	0.076	0.193
14/15	A	-0.052	0.076	0.227
	B	-0.064	0.075	0.213
15/15	-	-0.026	0.068	0.245

Table S5. Lists of the adsorption energies (E_{ads}) of water on the Ni_3P_4-terminated Ni_5P_4(0001) surface with two different adsorption sites; the distances from the O atom of H_2O to the adsorption sites (*d*(O-P/Ni)); the distances from the H atom of H_2O to the adsorption sites (*d*(H-P/Ni)); and the O-H bond lengths (*d*(O-H)) after H_2O adsorption on the two sites of the surface.

Adsorption site	E_{ads}/eV	*d*(O-P/Ni)/ Å	*d*(H-P/Ni)/ Å	*d*(O-H)/ Å
P_3-hollow	-0.183	3.097	2.728	0.977
Ni_3-hollow	-0.289	3.248	2.748	0.981

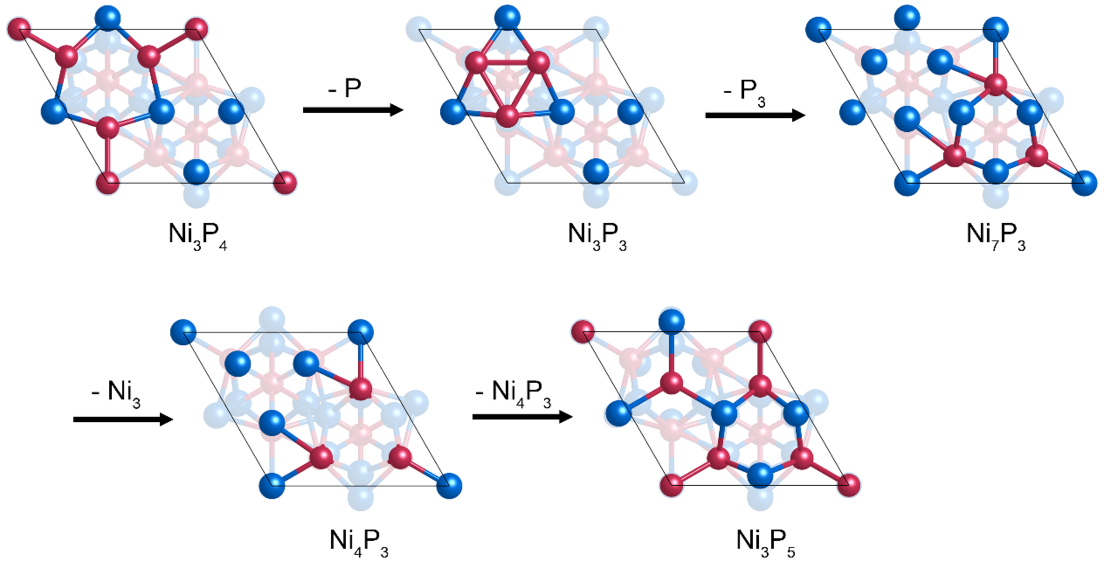

Figure S1. Schematic illustration of generating five possible surface terminations of Ni_5P_4 by removing the outermost atoms or atomic layers from the surface in the [0001] direction. The blue and red spheres represent Ni and P atoms, respectively.

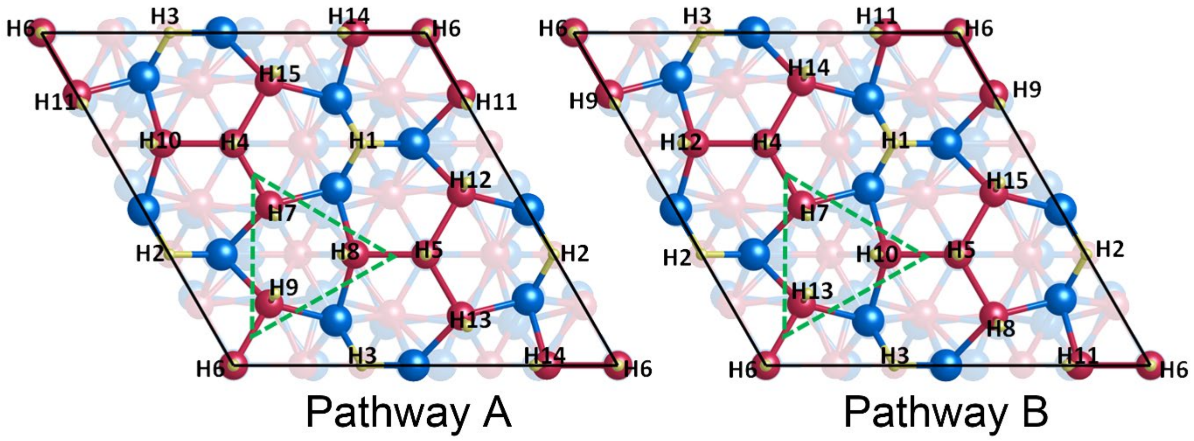

Figure S2. Schematic illustration of the two pathways (A and B) along which the H coverage increases on the Ni_3P_4-terminated $Ni_5P_4(0001)$ surface.

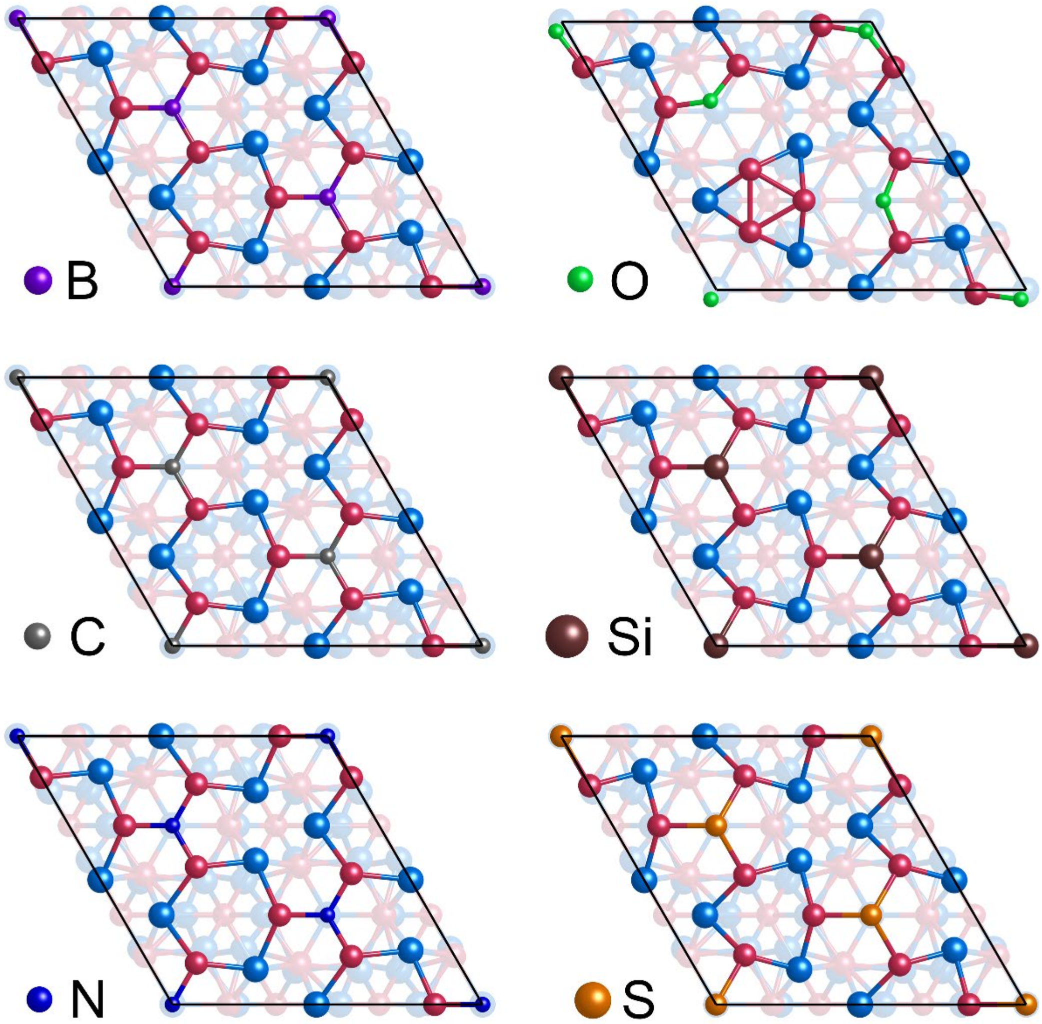

Figure S3. Optimized geometries of the Ni_3P_4-terminated $Ni_5P_4(0001)$ surface doped by different non-metal atoms.

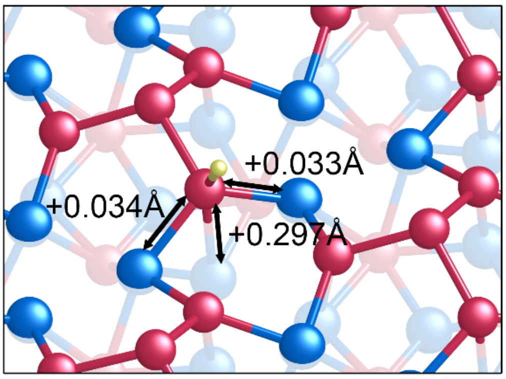

Figure S4. Schematics of the increases in the lengths of the Ni-P bonds around the hollow-P atom after H adsorption.

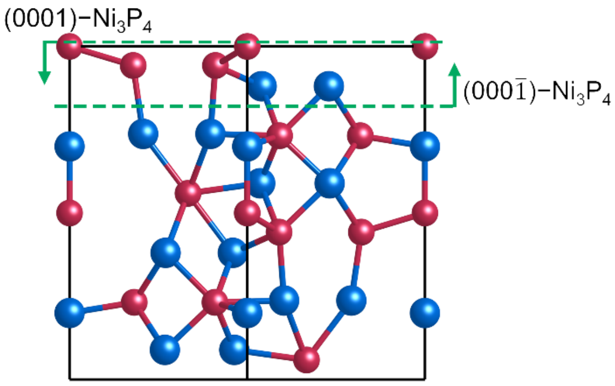

Figure S5. Schematic illustration of the generation of (0001)-Ni_3P_4 and (000$\bar{1}$)-Ni_3P_4 surfaces and their structural difference along the [0001] direction.

REFERENCES

(1) Wexler, R. B.; Martirez, J. M. P.; Rappe, A. M. Stable Phosphorus-Enriched (0001) Surfaces of Nickel Phosphides. *Chem. Mater.* **2016**, *28*, 5365–5372.

(2) Babizhetskyy, V.; Kotur, B.; Oryshchyn, S.; Zheng, C.; Kneidinger, F.; Leber, L.; Simson, C.; Bauer, E.; Michor, H. Crystal and Electronic Structure and Physical Properties of Ni_5P_4. *Solid State Commun.* **2013**, *164*, 1–5.

(3) Ledendecker, M.; Schlott, H.; Antonietti, M.; Meyer, B.; Shalom, M. Experimental and Theoretical Assessment of Ni-Based Binary Compounds for the Hydrogen Evolution Reaction. *Adv. Energy Mater.* **2017**, *7*, 1601735.
